# Association of Vitamin E Levels with Metabolic Syndrome, and MRI-Derived Body Fat Volumes and Liver Fat Content

**DOI:** 10.3390/nu9101143

**Published:** 2017-10-18

**Authors:** Sabina Waniek, Romina di Giuseppe, Sandra Plachta-Danielzik, Ilka Ratjen, Gunnar Jacobs, Manja Koch, Jan Borggrefe, Marcus Both, Hans-Peter Müller, Jan Kassubek, Ute Nöthlings, Tuba Esatbeyoglu, Sabrina Schlesinger, Gerald Rimbach, Wolfgang Lieb

**Affiliations:** 1 Institute of Epidemiology, Christian-Albrechts University of Kiel, 24105 Kiel, Germany; sabina.waniek@epi.uni-kiel.de (S.W.); romina.digiuseppe@epi.uni-kiel.de (R.d.G.); sandra.plachta-danielzik@epi.uni-kiel.de (S.P.-D.); ilka.ratjen@epi.uni-kiel.de (I.R.); jacobs@popgen.de (G.J.); mkoch@hsph.harvard.edu (M.K.); 2 Biobank PopGen, University Hospital Schleswig-Holstein, Campus Kiel, 24105 Kiel, Germany; 3 Department of Nutrition, Harvard T.H. Chan School of Public Health, Boston, MA 02115, USA; 4 Institute of Diagnostic and Interventional Radiology, University Hospital Cologne, 50937 Cologne, Germany; jan.borggrefe@uk-koeln.de; 5 Department of Radiology and Neuroradiology, University Hospital Schleswig-Holstein, Campus Kiel, 24105 Kiel, Germany; Marcus.both@uksh.de; 6 Department of Neurology, University of Ulm, 89081 Ulm, Germany; hans-peter.mueller@uni-ulm.de (H.-P.M.); jan.kassubek@uni-ulm.de (J.K.); 7 Department of Nutrition and Food Science, University of Bonn, 53113 Bonn, Germany; noethlings@uni-bonn.de; 8 Institute of Human Nutrition and Food Science, Christian-Albrechts University of Kiel, 24118 Kiel, Germany; tuba.esatbeyoglu@mri.bund.de (T.E.); rimbach@foodsci.uni-kiel.de (G.R.); 9 Institute for Biometrics and Epidemiology, German Diabetes Center (DDZ) at Heinrich Heine University Düsseldorf, 40225 Düsseldorf, Germany; sabrina.schlesinger@DDZ.uni-duesseldorf.de

**Keywords:** vitamin E, α- and γ-tocopherol, metabolic syndrome, body fat volumes, liver fat content

## Abstract

We aimed to relate circulating α- and γ-tocopherol levels to a broad spectrum of adiposity-related traits in a cross-sectional Northern German study. Anthropometric measures were obtained, and adipose tissue volumes and liver fat were quantified by magnetic resonance imaging in 641 individuals (mean age 61 years; 40.6% women). Concentrations of α- and γ-tocopherol were measured using high performance liquid chromatography. Multivariable-adjusted linear and logistic regression were used to assess associations of circulating α- and γ-tocopherol/cholesterol ratio levels with visceral (VAT) and subcutaneous adipose tissue (SAT), liver signal intensity (LSI), fatty liver disease (FLD), metabolic syndrome (MetS), and its individual components. The α-tocopherol/cholesterol ratio was positively associated with VAT (β scaled by interquartile range (IQR): 0.036; 95%Confidence Interval (CI): 0.0003; 0.071) and MetS (Odds Ratio (OR): 1.83; 95% CI: 1.21–2.76 for 3rd vs. 1st tertile), and the γ-tocopherol/cholesterol ratio was positively associated with VAT (β scaled by IQR: 0.066; 95% CI: 0.027; 0.104), SAT (β scaled by IQR: 0.048; 95% CI: 0.010; 0.087) and MetS (OR: 1.87; 95% CI: 1.23–2.84 for 3rd vs. 1st tertile). α- and γ-tocopherol levels were positively associated with high triglycerides and low high density lipoprotein cholesterol levels (all P_trend_ < 0.05). No association of α- and γ-tocopherol/cholesterol ratio with LSI/FLD was observed. Circulating vitamin E levels displayed strong associations with VAT and MetS. These observations lay the ground for further investigation in longitudinal studies.

## 1. Introduction

Metabolic conditions like metabolic syndrome (MetS), fatty liver disease (FLD), and obesity have been linked to increased inflammation and oxidative stress [1,2,3]. Vitamin E is a lipid-soluble vitamin, encompassing different tocopherols (α-, β-, γ-, and δ-tocopherol) with important anti-oxidative and potentially anti-inflammatory functions [4,5]. In a recent randomized trial [6], vitamin E administration over 96 weeks performed better than pioglitazone and better than placebo in patients with non-alcoholic steatohepatitis (NASH). It is, therefore, conceivable that vitamin E levels are altered in patients with MetS or FLD and that vitamin E levels are correlated with other adiposity-related traits.

Previous clinical and epidemiologic studies on the association of circulating vitamin E levels with different anthropometric adiposity measures (e.g., waist circumference, body mass index (BMI)) [7,8,9,10,11], the MetS [12,13,14,15,16], and NASH [17,18,19] produced partially conflicting results. Among the different fat depots, subcutaneous (SAT) and, particularly, visceral adipose tissue (VAT) are considered relevant for metabolic conditions, such as MetS. Whether vitamin E levels are associated with MetS, FLD and other adiposity measures, including SAT and VAT, as determined by magnetic resonance imaging (MRI), is unknown.

Therefore, we aimed to relate circulating levels of α- and γ-tocopherol to a broad spectrum of adiposity-related traits in a community-based sample from Northern Germany. Specifically, we assessed the associations of plasma α- and γ-tocopherol levels with MetS and its individual components, MRI-determined VAT, SAT, and liver signal intensity (LSI), as well as with the presence or absence of FLD. We hypothesize that vitamin E levels are altered in individuals with MetS and that vitamin E levels are associated with VAT, SAT, and liver fat, as determined by MRI.

## 2. Materials and Methods

### 2.1. Study Sample

Between 2005 and 2007, a total of 1316 individuals from Northern Germany were recruited by the PopGen biobank [20]. Specifically, the sample consisted of 747 individuals who were identified through official population registries and from 569 blood donors. The first follow-up examination, conducted between 2010 and 2012, was attended by 952 individuals, who received a physical examination conducted by trained personnel and provided blood and urine samples. Furthermore, all participants filled-in a standardized questionnaire on demographic and health-related characteristics (including dietary intake, education, smoking status, and physical activity) and medical history [20,21]. A subsample of participants (*n* = 641) agreed to undergo whole-body MRI. From these participants concentrations of circulating α- and γ-tocopherol levels were measured. Thus, the association between plasma vitamin E concentrations and MetS was investigated in 641 individuals. A total of 91 individuals had to be excluded from the analyses related to MRI phenotypes because of insufficient imaging quality (*n* = 35), non-adherence to the MRI breathing protocol (*n* = 40), and missing information on quality of MRI assessment (*n* = 16). Further, individuals with self-reported liver disease (hepatitis A, B, C, or D virus infection, hemochromatosis, autoimmune liver disease, or liver cirrhosis (*n* = 29)) were excluded. Thus, the association between circulating vitamin E biomarkers and VAT and SAT was assessed in 591 individuals and the association with liver fat and FLD was evaluated in 571 individuals.

The study has been approved by the Ethics Committee of the Medical Faculty of the Christian-Albrechts University Kiel. Written informed consent was obtained from all study participants.

### 2.2. Physical Examination and Standardized Questionnaires

Weight and height were measured with the participant wearing light clothing and no shoes, and 2.0 kg were subtracted to correct for the remaining clothes. BMI was calculated as body weight (kg)/height (m^2^). Waist circumference was measured at the midpoint between the lower ribs and iliac crest on the anterior axillary line in a resting expiratory position. After the participants had rested 5 min in a sitting position, blood pressure was measured twice (2 min interval) using a sphygmomanometer [22]. Dietary intake, including information on alcohol consumption during the last 12 months, was assessed by a self-administered semi-quantitative 112-item food-frequency questionnaire (FFQ) designed and validated especially for the German population [23]. The German Food Code and Nutrient Data Base (version II.3) was used to determine energy intake and was provided by the Department of Epidemiology of the German Institute of Human Nutrition Potsdam Rehbrücke [24]. Participants were asked to report their use of vitamin E supplements in the FFQ.

### 2.3. Assessment of SAT, VAT, and Liver Fat Using MRI

Liver fat and adipose tissue (AT) volumes (defined as VAT and SAT) were measured by MRI using a 1.5-T scanner (Magnetom Avanto; Siemens Medical solution, Erlangen, Germany), as described in detail elsewhere [25,26,27]. VAT was determined as the sum of VAT voxels from the top of the liver to the femoral heads inside the abdominal muscular wall as anatomical border and SAT was determined as the sum of AT voxels underneath the skin layer surrounding the abdomen from the top of the liver to the femur heads. To obtain the volumes (in dm^3^) of VAT and SAT the voxel size (3.9 × 2 × 8 mm^3^) was multiplied by the number of voxels [26].

Liver fat was quantified as relative LSI difference of the liver on out-of-phase compared with in-phase images in arbitrary units. Both in- and out- of phase images were acquired during a breath hold by using axial T1-weighted gradient echo sequences. Signal intensities were obtained by measuring the average of three circular regions of interest in the liver parenchyma [27].

### 2.4. Definitions

Hypertension was defined as systolic blood pressure ≥140 mmHg or diastolic blood pressure ≥90 mmHg, or self-reported hypertension history or use of antihypertensive medication.

MetS was defined according to the harmonized criteria [28] and was considered present when at least three of the following five criteria were met: (1) elevated triglyceride concentration (≥150 mg/dL); (2) reduced high density lipoprotein (HDL)-concentration (<40 mg/dL in men or <50 mg/dL in women); (3) elevated blood pressure (systolic blood pressure ≥130 mmHg and/or diastolic blood pressure ≥85 mmHg or anti-hypertensive treatment); (4) dysglycaemia, defined as elevated plasma fasting glucose (≥100 mg/dL) or anti-diabetic treatment; and (5) abdominal obesity (waist circumference ≥94 cm for men and ≥80 cm for women). In the present definition, information about triglyceride-lowering and HDL-increasing medications were not included because this information was not available in detail in our sample. Type 2 diabetes was defined as glycated hemoglobin (HbA1c) ≥6.5% (48 mmol/mol) or fasting glucose ≥126 mg/dL, or use of anti-diabetic medication or self-report physician diagnosis. FLD was defined as log liver signal intensity ≥3.0 according to a cut-off, which corresponds to the maximum Youden Index and was derived using spectroscopically determined FLD (liver fat ≥ 5.56%) as the reference method [27,29].

Total physical activity was defined as the reported frequency (hour/week) of different activities (leisure and working-time) [30], multiplied by the corresponding metabolic equivalent (MET)-value, and summed up for all activities [22,31]. Participants were classified into 3 categories to determine smoking status: no-smokers if they had never smoked; former smokers if they had smoked in the past and quit smoking more than 1 year ago; and current smokers if they were currently smoking 1 or more cigarettes per day. Participants were categorized according to the level of education into three categories: low (≤9 years), middle (10 years), or high (≥11 years).

### 2.5. Laboratory Analyses

Fasting blood (EDTA whole-blood and lithium heparin) samples were obtained from participants in a sitting position. All blood samples were centrifuged, aliquoted, and stored at −80 °C. In fresh blood samples, concentrations of C-reactive protein (CRP), triglycerides, HDL-cholesterol, and total cholesterol were analyzed by enzymatic colorimetry (Roche Diagnostic, Mannheim, Germany); the concentration of glucose was determined by using enzymatic ultraviolet tests (Roche Diagnostic, Mannheim, Germany), and HbA1c concentrations were determined by using high performance liquid chromatography (HPLC) and photometric detection (Bio-Rad Laboratories, Munich, Germany) in EDTA plasma.

Laboratory blood analyses were performed in the laboratory for clinical chemistry of the University Hospital Schleswig-Holstein, Campus Kiel in Germany.

The Institute of Human Nutrition and Food Science at the Christian-Albrechts-University of Kiel in Germany measured plasma vitamin E (α- and γ-tocopherol) levels using a HPLC with fluorescence detection. An external standard curve was used to quantify vitamin E concentrations using a Jasco HPLC system (Jasco GmbH Deutschland, Gross-Umstadt, Germany; equipped with an autosampler (Jasco AS-2057), pump (PU-2080), ternary gradient unit (LG-2080-02), 3 line degasser (DG-2080-53), and fluorescence detector (FP2020 Plus)) with a Waters Spherisorb ODS-2,3 µm column (100 × 4.6 mm) using methanol:water (98:2, *v/v*) as mobile phase. The fluorescence detector operated an excitation wavelength of 290 nm and emission wavelength of 325 nm. The flow rate of the mobile phase was set at 1.2 mL/min. Duplicate measurements were performed and the injection volume was set at 40 µL. Plasma (50 µL) was homogenized in 2 mL 1% ascorbic acid (in ethanol), 700 µL deionised water, 50 µL 0.1% butylated hydroxytoluol (in ethanol), and 2 mL n-hexane were prepared for analysing the samples. The samples were centrifuged (1000× *g* for 5 min at 4 °C). After separating the phases, 1000 µL of the upper phase was dried under vacuum in a RC-1010 centrifugal evaporator (Jouan, Saint-Herblain, France). The samples were re-suspended in 200 µL mobile phase (methanol:water, 98:2, *v/v*) [32]. The coefficients of variation for α- and γ-tocopherol were 1.05% and 1.29%, respectively.

### 2.6. Statistical Analyses

Some few missing values of covariates were replaced by a simple imputation, as follows: When values of categorical variables were missing, they were replaced by the most commonly observed category of that respective variable (*n* = 10). Normally distributed continuous missing variables were imputed by the respective mean and skewed variables by the sex-specific median (*n* = 2). Detailed information of missing covariates are provided in Supplementary Materials Table S1.

CRP values below 0.9 mg/dL (detection limit) were assigned a value equal to the half of the detection limit (*n* = 247). Values of γ-tocopherol (*n* = 14, respectively) were imputed by the lowest γ-tocopherol concentration measured in our sample. Detailed information of missing covariates are provided in Supplementary Materials Table S1.

Because vitamin E is bound to lipoproteins in the blood stream [33], cholesterol-adjusted α- and γ-tocopherol levels (μmol/mmol) were calculated by dividing α- and γ-tocopherol concentrations (μmol/L) by total cholesterol (mmol/L) [34].

We performed the following analyses: For descriptive purposes, anthropometric, lifestyle, and clinical factors were compared across tertiles of the α- and γ-tocopherol/cholesterol ratios. Differences in median of continuous variables were tested by using Wilcoxonń s rank-sum test, and differences in categorical variables were assessed by using a chi-square test.

Restricted cubic splines analyses displayed linear associations between vitamin E biomarkers and continuous (VAT, SAT, LSI) and binary (MetS, and FLD) outcomes. Third, linear and logistic regression models were used to relate circulating vitamin E (α- and γ-tocopherol/cholesterol ratio, each biomarker considered separately) levels to continuous outcomes (VAT, SAT, LSI) and binary outcomes (MetS, individual components of MetS, FLD), respectively. In linear regression models, both α- and γ-tocopherol levels were scaled to their interquartile range (IQR) and β coefficients interpreted as comparing VAT, SAT, and LSI values of a person with a typical “high” α- or γ-tocopherol value to a person with a typical “low” value.

Adjusted means of VAT, SAT, and LSI were calculated by general linear models, respectively. We ran age- and sex-adjusted, as well as multivariable-adjusted, models which included, based on literature research [12,13], age (continuous in years) and sex, education (low, medium, high), physical activity (continuous in MET-hour/week), smoking status (never, current, former), vitamin E supplementation (yes, no), alcohol intake (continuous in g/day), and total energy intake (continuous in kJ/day) as potential confounders. Furthermore, the models with continuous VAT and SAT as outcome variables were additionally adjusted for BMI (continuous in kg/m^2^) to assess whether VAT and SAT were associated with circulating vitamin E levels independent of BMI. Individual components of MetS were adjusted for each of the other four criteria for the MetS.

Potential interactions of age, sex, and vitamin E supplementation with each metabolic outcome (VAT, SAT, LSI, MetS, individual components of MetS, FLD) were tested by including multiplicative interaction terms into the regression models. In a sensitivity analysis, we excluded vitamin E supplement users and we related α- and γ-tocopherol/cholesterol ratio levels (each biomarker considered separately) to each selected metabolic outcome (Supplementary Materials Tables S4–S6). Furthermore, we excluded individuals who reported a consumption of alcohol more than 20 g/day (*n* = 134) when examining the association of α- and γ-tocopherol/cholesterol ratio with FLD.

Categorical variables with more than two categories were included as indicator variables. P_trend_ was calculated across tertiles using median values of α- and γ-tocopherol/cholesterol ratio within each tertile and we used these values as continuous variables.

All statistical tests were two-sided and considered to be significant when *p* values < 0.05. All analyses were performed with SAS 9.4 (SAS Institute, Cary, NC, USA).

## 3. Results

### 3.1. General Characteristics

General characteristics of the study sample according to tertiles of the α- and γ-tocopherol/cholesterol ratio are depicted in Table 1 and Table 2, respectively. Triglycerides levels were higher in the 3rd tertile compared to the 1st tertile of the α-tocopherol/cholesterol ratio. Furthermore, the proportion of individuals with MetS was higher in the upper tertiles of the α-tocopherol/cholesterol ratio. Similarly, triglycerides levels were higher in the 3rd tertile compared to the 1st tertile of the γ-tocopherol/cholesterol ratio. Furthermore, BMI and waist circumference, CRP levels, as well as VAT and SAT were higher in the 3rd tertile compared to the 1st tertile of the γ-tocopherol/cholesterol ratio. In addition, the prevalence of the MetS and of diabetes rose with tertiles of the γ-tocopherol/cholesterol ratio. The proportion of vitamin E supplement users was highest in the bottom tertile of γ-tocopherol/cholesterol ratio.

### 3.2. Association of α-Tocopherol/Cholesterol Ratio with Metabolic Traits

In multivariable-adjusted linear regression models, plasma α-tocopherol/cholesterol ratio displayed a statistically significant association with VAT (β scaled by IQR: 0.036; 95% Confidence Interval (CI): 0.0003; 0.071), including a model additionally adjusted for BMI (β scaled by IQR: 0.026; 95% CI: 0.002; 0.050) (Table 3). Furthermore, consistent associations of plasma α-tocopherol/cholesterol ratio with the MetS were observed (Odds Ratio (OR): 1.83; 95% CI: 1.21–2.76 for 3rd vs. 1st tertile; P_trend_ = 0.003) (Table 5), driven by positive associations with high triglycerides (OR: 3.02; 95% CI: 1.80–5.06 for 3rd vs. 1st tertile; P_trend_ < 0.0001) and low HDL-cholesterol levels (OR: 2.52; 95% CI: 0.97–6.56 for 3rd vs. 1st tertile; P_trend_ = 0.033) (Supplementary Materials Table S2). The α-tocopherol/cholesterol ratio was neither associated with SAT, nor with LSI, modeled as a continuous or binary trait (FLD) (Table 3 and Table 5).

### 3.3. Association of γ-Tocopherol/Cholesterol Ratio with Metabolic Traits

In multivariable-adjusted linear and logistic regression models, plasma γ-tocopherol/cholesterol ratio showed statistically significant associations with VAT (β scaled by IQR: 0.066; 95% CI: 0.027; 0.104), SAT (β scaled by IQR: 0.048; 95% CI: 0.010; 0.087), and the MetS (OR: 1.87; 95% CI: 1.23–2.84 for 3r^d^ vs. 1st tertile; P_trend_ = 0.004) (Table 4 and Table 5). The association with VAT persisted upon additional adjustment for BMI (β scaled by IQR: 0.037; 95% CI: 0.011; 0.063), whereas adding BMI to the model rendered the association of γ-tocopherol/cholesterol ratio with SAT statistically non-significant (β scaled by IQR: 0.015; 95% CI: −0.006; 0.037) (Table 4). Regarding the individual components of the MetS, the γ-tocopherol/cholesterol ratio was positively related to hypertriglyceridemia (OR: 1.81; 95% CI: 1.08–3.06 for 3rd vs. 1st tertile; P_trend_ = 0.014) and low HDL-cholesterol levels (OR: 4.67; 95% CI: 1.42–15.41 for 3rd vs. 1st tertile; P_trend_ = 0.018) in multivariable-adjusted models (Supplementary Materials Table S3).

No association of γ-tocopherol/cholesterol ratio with LSI (continuous trait) or FLD (binary trait) was observed (Table 4 and Table 5).

### 3.4. Assessment of Interactions and Sensitivity Analyses

No significant interactions between circulating α- and γ-tocopherol/cholesterol concentrations and age, sex, or vitamin E supplementation in relation to each selected outcome were observed (*p* > 0.05 for all). In a sensitivity analysis, excluding vitamin E supplement users, the magnitude and the direction of the associations were essentially unchanged (Supplementary Materials Tables S4–S6).

With respect of the association of vitamin E levels with FLD, the results were essentially unchanged after excluding individuals with an alcohol consumption of more than 20 g/day. In multivariable-adjusted models, the α- and γ-tocopherol/cholesterol ratio were not statistically significantly related to the probability of having FLD (OR: 1.09; 95% CI: 0.66–1.80 for 3rd vs. 1st tertile; P_trend_ = 0.268 and OR: 1.51; 95% CI: 0.94–2.55 for 3rd vs. 1st tertile; P_trend_ = 0.239, respectively).

## 4. Discussion

### 4.1. Principal Observations

In a community-based sample from Northern Germany, the α-tocopherol/cholesterol ratio and the γ-tocopherol/cholesterol ratio were positively associated with VAT, SAT, MetS, and its components, high triglycerides and low HDL-cholesterol levels. No significant associations were observed when α- and γ-tocopherol/cholesterol ratios were studied in relation to LSI or FLD.

### 4.2. In the Context of the Published Literature

#### 4.2.1. Vitamin E Levels and Measures of Adiposity and Adipose Tissue Volumes

We observed a consistent association of α- and γ tocopherol/cholesterol ratio with VAT; the γ-tocopherol/cholesterol ratio was also associated with SAT. This is in line with several prior studies that reported positive associations of circulating vitamin E levels with adiposity measures (e.g., BMI, waist circumference, waist-to-hip ratio, and waist-to-height ratio) [7,8,9]. For example, in a subsample of participants in the Women’s Health Initiative (*n* = 2672), circulating γ-tocopherol levels were positively and strongly associated with BMI, waist circumference, and waist-to-height ratio, while α-tocopherol levels were only positively associated with waist-to-hip ratio [7]. Likewise, Chai et al. [8] reported in 180 premenopausal women that γ-tocopherol levels were significantly higher in obese individuals, whereas α-tocopherol levels did not differ among BMI subgroups.

With respect to α-tocopherol, Wallström et al. [9] reported that serum levels of α-tocopherol were positively related to central adiposity (defined as waist circumference and waist-to-hip ratio), but BMI was only associated with α-tocopherol in men. Body fat percentage (determined by bioelectrical impedance analysis), however, was not significantly associated with vitamin E [9]. Interestingly, in a subsample of healthy postmenopausal women (*n* = 48), α-tocopherol was identified as predictor of MRI-determined total fat [35]. By contrast, in a sample of 580 women, no association of vitamin E levels with measures of adiposity (BMI, waist circumference, waist-to-height ratio, visceral adiposity, and total body fat) determined by dual-energy x-ray absorptiometry was observed [10].

#### 4.2.2. Vitamin E Levels and the Metabolic Syndrome

We observed consistent positive associations of α- and γ-tocopherol/cholesterol ratio levels with MetS in different multivariable-adjusted statistical models. These associations were driven by a positive association with low HDL-cholesterol levels and high triglycerides levels. The association of vitamin E levels with lipid traits is biologically plausible because the lipid-soluble vitamin E is transported in the blood by lipoproteins [33].

In contrast to our observations, in one study, lower levels of plasma α-tocopherol levels were reported in individuals with MetS (*n* = 182) compared to healthy adults (*n* = 91) [14]. In a much larger sample from the 2001–2006 National Health Examination Survey (NHANES; *n* = 3008), no association of vitamin E levels with MetS was reported. However, in further analyses, the authors observed that vitamin E concentrations were significantly positively related to the number of MetS criteria [12], which lends some support to our results.

Regarding individual components of MetS, vitamin E controlled for lipids showed a positive association with hypertriglyceridemia but not with low HDL-cholesterol levels in NHANES samples [12,13]. A possible explanation for the discrepancies might be that we considered each biomarker separately (α- and γ-tocopherol/cholesterol ratio, respectively), whereas in the other studies vitamin E was defined as the sum of α- and γ-tocopherol. Additionally, we only had a low number of individuals (*n* = 37) with low HDL-cholesterol levels, whereas both of the other studies [12,13] included more participants (*n* = 4322 and *n* = 8465, respectively).

#### 4.2.3. Vitamin E Levels, Fatty Liver Disease, and Liver Fat Content

The association of vitamin E levels with NASH has been assessed in some prior clinical settings with rather small samples sizes [17,18,19]. One study [17] reported, on average, higher serum vitamin E levels in 43 patients with histologically proven NASH as compared to 33 healthy controls. In two other studies [18,19], however, vitamin E levels were lower in biopsy-proven NASH patients (*n* = 50 and *n* = 29, respectively) than in controls (*n* = 40 and *n* = 10, respectively).

Regarding liver fat content in postmenopausal healthy women, α-tocopherol was identified as a predictor of MRI-determined liver fat, along with other biomarkers [35]. However, this study was based on a rather small sample (*n* = 48) of postmenopausal women, with lack of generalizability to other women and to men [35]. We expand these analyses by assessing in a much larger sample (*n* = 571) from the general population, including men and women, the associations of circulating α- and γ-tocopherol/cholesterol ratio levels with MRI-determined LSI, a proxy for liver fat, modeled on a continuous scale and as a binary trait (FLD).

Yet, albeit FLD is commonly subdivided into non-alcoholic FLD and alcoholic FLD [36], others questioned such a distinction, e.g., because of, in part, similar pathological findings in alcoholic and non-alcoholic FLD, in part overlapping pathophysiological features, sharing of alcohol and other risk factors for FLD in a substantial fraction of the population, and a lack of a consensus regarding harmless alcohol consumption [37]. Furthermore, both non-alcoholic FLD and alcoholic FLD have been associated with premature atherosclerosis, and these findings support the paradigm that steatosis might be a precursor of an increased cardiovascular risk [38]. Therefore, considering FLD as a complex, multifactorial condition [37], we did not distinguish between alcoholic and non-alcoholic FLD but focused on MRI-derived LSI as a proxy for liver fat content.

Interestingly, vitamin E therapy (800 UI per day) for 96 weeks performed better than pioglitazone and placebo in a randomized trial in patients with NASH [6]. The primary endpoint of the study was a histological improvement of NASH features [6].

However, in contrast to the studies mentioned above, we observed no association between vitamin E levels and LSI or FLD in our sample. One potential explanation is that vitamin E levels are altered preferentially in patients with advanced liver disease [39], but not in relatively healthy men and women from the general population with rather modest alterations in liver fat, a premise that merits further investigations.

### 4.3. Potential Mechanisms for the Observed Associations

Our data suggest that circulating vitamin E (α- and γ-tocopherol/cholesterol ratio) levels are positively associated with MetS and MRI-determined body fat volumes (particularly VAT).

Circulating vitamin E levels are affected by several factors: Dietary vitamin E is absorbed in the gastro-intestinal system (the efficiency of vitamin E absorption is widely variable, ranging from 20–80%) and transported via chylomicrons to the liver [40]. Taken up by the liver, α-tocopherol has several possible metabolic pathways. The hepatic α-tocopherol transfer protein (α-TTP) is the major regulator for maintaining normal plasma α-tocopherol concentrations [41]. γ-tocopherol has much less affinity (α-tocopherol = 100%, γ-tocopherol = 9% [42]) for α-TTP and is largely metabolized in the liver and secreted in the bile [43]. Experimental evidence indicates that α-TTP activity is modulated by oxidative stress potentially influencing vitamin E status [44,45,46,47].

α-TTP can facilitate α-tocopherol transfer to very low density lipoproteins (VLDL), whereas the underlying mechanism is still not understood, and facilitate its return to the liver [40]. It is suggested that VLDL is enriched with α-tocopherol to a lesser extent in MetS compared to healthy adults and therefore transported in VLDL to a lesser degree to extrahepatic tissues in individuals with MetS because of a slower α-tocopherol catabolism in MetS compared to healthy adults [16,48]. Therefore, it might be that the disappearance of α-tocopherol from plasma is slower in individuals with MetS [16,48], which would explain the positive association of plasma α-tocopherol levels with MetS as observed in our analyses.

Looking at the excretion, α-tocopherol can be secreted in bile for fecal excretion, but it is not known if this pathway is altered in individuals with MetS [48]. Interestingly, data from a recent study [48] indicate that MetS may inhibit the hepatic metabolism of α-tocopherol to the α-tocopherol metabolite α-carboxyethyl hydroxychromanol (CEHC) (secreted in bile for elimination via feces or excreted via urine [43]). In a recent clinical trial, Traber et al. [48] observed that individuals with MetS (*n* = 10) excrete less vitamin E (lower amounts of α- and γ-CEHC were detected in the urine) as compared to healthy adults (*n* = 10). The authors speculated that individuals with MetS might need more vitamin E because of increased oxidative and inflammatory stressors, thereby suggesting they had increased requirements for α-tocopherol and therefore retained more vitamin E compared to healthy adults [48]. We observed that α- and γ-tocopherol/cholesterol ratios were more strongly and positively associated with VAT than with SAT. Indeed, it is known that adipose tissue, as an endocrine organ, contains a large number of pro-inflammatory cytokines including tumor necrosis factor-α, interleukin (IL)-1β, and IL-6-promoting inflammatory response and oxidative stress [49,50]. Of note, VAT has been shown to release more inflammatory markers (e.g., two to three times more IL-6) than SAT [50]; a rise in concentration of inflammatory markers could be responsible for increased oxidative stress leading to higher vitamin E levels as a compensatory mechanism. Furthermore, in our study, adjustment for BMI rendered the association of γ-tocopherol/cholesterol with SAT statistically non-significant, but not the association with VAT. This might be explained by the fact that BMI is more strongly correlated with SAT than with VAT [51].

### 4.4. Strengths and Limitation

Strengths of the present study include the assessment of VAT, SAT and liver fat by MRI in a moderate-sized sample from the general population, the measurement of vitamin E in plasma, and the detailed assessment of covariates. The following limitation merits consideration. The cross-sectional study design precludes causal inferences, because exposure and outcome were assessed at the same time. Furthermore, we cannot completely rule out reverse causality. Besides, the cross-sectional study design and the small regression coefficients observed for the associations of VAT and SAT, with both α- and γ-tocopherol values, limit our ability to quantify and translate the observed associations into clinical meaningful findings. Moreover, we had no information about why individuals were taking vitamin E supplements and about the use of statins. However, we did have self-reported information regarding the use of lipid-lowering medications for a subsample (*n* =305).

In summary, we observed significant associations of circulating vitamin E concentrations with MetS and MRI-determined body fat volumes (particularly VAT). Further investigations of longitudinal relationships between α- and γ-tocopherol levels and metabolic conditions and liver fat are warranted.

## Figures and Tables

**Table 1:** General characteristics of the PopGen control study population (*n* = 641) according to tertiles (T) of α-tocopherol/cholesterol ratio.

Characteristics	Tertiles α-Tocopherol/Cholesterol Ratio						*p*
	T1 (*n* = 213)		T2 (*n* = 214)		T3 (*n* = 214)		
Median α-tocopherol/cholesterol ratio (IQR), µmol/mmol	4.63	(4.25–4.88)	5.53	(5.36–5.72)	6.74	(6.33–7.59)	
Men, %	55.9		61.7		60.8		0.422
Age, years	63.0	(56.0–70.0)	61.5	(54.0–71.0)	62.0	(51.0–71.0)	0.411
Body mass index, kg/m^2^	26.6	(23.3–29.8)	26.7	(24.8–29.4)	26.7	(24.6–29.2)	0.633
Waist circumference, cm							
Men	100.0	(92.8–107.4)	100.2	(92.7–105.9)	99.4	(93.5–106.8)	0.956
Women	87.1	(78.5–96.4)	88.5	(83.2–97.4)	92.4	(80.2–99.6)	0.199
Systolic blood pressure, mm/Hg	139.0	(127.5–150.0)	140.0	(125.0–150.0)	138.3	(125.0–150.0)	0.856
Diastolic blood pressure, mm/Hg	85.0	(80.0–90.0)	85.0	(80.0–90.0)	82.3	(80.0–90.0)	0.341
Prevalent hypertension, %	68.1		71.0		67.8		0.723
Current smokers, %	10.1		9.8		12.2		0.640
Physical activity, MET-hour/week	98.3	(61.5–141.6)	84.2	(54.8–120.1))	90.0	(59.3–131.7)	0.074
High education (≥11 years), %	29.1		40.7		37.9		0.143
Alcohol consumption, g/day	8.67	(2.76–17.0)	8.58	(4.09–17.95)	10.96	(4.15–20.05)	0.114
Vitamin E supplementation, %	5.6		6.5		10.3		0.154
Prevalent diabetes, %	8.9		8.9		14.5		0.099
Metabolic syndrome, %	36.6		36.0		48.1		0.016
C-reactive protein, mg/dL	1.10	(0.45–2.60)	1.20	(0.45–2.40)	1.40	(0.45–2.20)	0.531
HDL-cholesterol, mg/dL	67.0	(56.0–82.0)	63.5	(54.0–76.0)	57.5	(49.0–72.0)	<0.0001
Triglycerides, mg/dL	96.0	(71.0–123.0)	104.0	(78.0–132.0)	123.0	(84.0–169.0)	<0.0001
Diabetes medication, % *	3.6		7.1		14.6		0.015
Lipid-lowering medication, % *	13.6		29.3		45.8		<0.0001
Fatty liver disease, % ^†^	38.9		38.5		40.3		0.928
Liver signal intensity ^†^	18.6	(14.9–23.4)	18.2	(15.0–22.1)	18.0	(14.5–24.7)	0.925
Visceral adipose tissue, dm^3 ‡^	3.70	(2.18–5.02)	3.90	(2.41–5.25)	3.94	(2.54–5.37)	0.478
Subcutaneous adipose tissue, dm^3 ‡^	5.91	(4.45–8.23)	6.45	(4.75–8.53)	6.10	(4.88–8.24)	0.546

Data are reported as percentages (%) or median and interquartile range (IQR). * *n* = 305, ^†^ *n* = 571, ^‡^ *n* = 591; MET: Metabolic equivalent; HDL: High density lipoprotein

**Table 2:** General characteristics of the PopGen control study population (*n* = 641) according to tertiles of γ-tocopherol/cholesterol ratio.

Characteristics	Tertiles (T) γ-Tocopherol/Cholesterol Ratio						*p*
	T1 (*n* = 213)		T2 (*n* = 214)		T3 (*n* = 214)		
Median γ-tocopherol/cholesterol ratio (IQR), µmol/mmol	0.16	(0.13–0.18)	0.24	(0.22–0.26)	0.35	(0.31–0.42)	
Men, %	60.06		57.9		59.8		0.851
Age, years	63.0	(55.0–71.0)	61.5	(55.0–71.0)	62.0	(54.0–69.0)	0.709
Body mass index, kg/m2	26.1	(23.4–28.9)	27.3	(24.8–29.6)	26.8	(24.9–30.7)	0.005
Waist circumference, cm							
Men	98.9	(91.5–105.3)	100.8	(93.5–108.3)	100.7	(94.6–106.9)	0.271
Women	85.3	(77.4–93.6)	89.0	(82.4–98.0)	91.8	(80.2–103.5)	0.002
Systolic blood pressure, mm/Hg	139.0	(126.5–150.0)	140.0	(125.0–150.0)	139.0	(127.5–150.0)	0.858
Diastolic blood pressure, mm/Hg	85.0	(80.0–90.0)	85.0	(80.0–90.0)	85.0	(80.0–90.0)	0.853
Prevalent hypertension, %	67.1		71.5		68.2		0.598
Current smokers, %	8.0		14.5		10.3		0.278
Physical activity, MET-hour/week	86.3	(58.8–130.0)	89.5	(59.8–138.2)	90.8	(56.8–125.4)	0.932
High education (≥11 years), %	40.9		31.3		35.5		0.168
Alcohol consumption, g/d	8.87	(3.20–16.79)	10.18	(3.82–20.3)	9.74	(4.0–20.13)	0.504
Vitamin E supplementation, %	14.6		2.8		5.1		<0.0001
Prevalent diabetes, %	8.0		8.9		15.4		0.026
Metabolic syndrome, %	32.9		41.6		46.3		0.017
C-reactive protein, mg/dL	1.0	(0.45–1.90)	1.30	(0.45–2.80)	1.40	(0.45–2.60)	0.009
HDL-cholesterol, mg/dL	66.0	(54.0–79.0)	62.0	(53.0–79.0)	60.0	(51.0–74.0)	0.023
Triglycerides, mg/dL	100.0	(76.0–131.0)	103.0	(72.0–133.0)	115.5	(80.0–158.0)	0.004
Diabetes medication, % *	4.0		3.9		17.0		0.0005
Lipid-lowering medication, % *	22.8		26.9		37.0		0.073
Fatty liver disease, % ^†^	37.4		36.7		43.7		0.303
Liver signal intensity ^†^	18.5	(14.7–22.4)	17.9	(14.5–24.1)	18.8	(14.8–24.2)	0.599
Visceral adipose tissue, dm^3 ‡^	3.55	(2.26–4.95)	3.82	(2.46–5.16)	4.15	(2.71–5.77)	0.013
Subcutaneous adipose tissue, dm^3 ‡^	5.85	(4.33–7.70)	6.33	(4.81–8.46)	6.30	(4.89–9.09)	0.018

Data are reported as percentages (%) or median and interquartile range (IQR). * *n* = 305, ^†^ *n* = 571, ^‡^ *n* = 591; MET: Metabolic equivalent; HDL: High density lipoprotein.

**Table 3:** Multivariable-adjusted means and 95% CI of VAT, SAT, and LSI according to tertiles of α-tocopherol/cholesterol ratio, and scaled by IQR.

Outcome	Tertiles (T) α-Tocopherol/Cholesterol Ratio			P_trend_	β Scaled by IQR and 95% CI
	T1	T2	T3		
N	196	199	196		
Median α-tocopherol/cholesterol ratio (IQR), µmol/mmol	4.49 (4.41–4.57)	5.53 (5.44–5.63)	7.18 (7.05–7.30)		
**VAT, dm^3^ (*n* = 591)**					
Model 1	2.99 (2.74–3.26)	3.20 (2.94–3.48)	3.29 (3.04–3.57)	0.056	0.035 (−0.002; 0.071)
Model 2	2.92 (2.63–3.26)	3.13 (2.82–3.47)	3.23 (2.93–3.57)	0.043	0.036 (0.0003; 0.071)
Model 3	3.09 (2.87–3.32)	3.31 (3.09–3.32)	3.34 (3.13–3.57)	0.016	0.026 (0.002; 0.050)
**SAT, dm^3^ (*n* = 591)**					
Model 1	6.07 (5.78–6.61)	6.38 (5.88–6.93)	6.32 (5.83–6.84)	0.437	0.025 (−0.011; 0.062)
Model 2	5.98 (5.38–6.66)	6.23 (5.62–6.91)	6.22 (5.64–6.87)	0.433	0.026 (−0.009; 0.062)
Model 3	6.36 (6.00–6.74)	6.64 (6.28–7.02)	6.46 (6.12–6.81)	0.572	0.015 (−0.004; 0.034)
N	190	191	190		
Median α-tocopherol/cholesterol ratio (IQR), µmol/mmol	4.50 (4.42–4.58)	5.54 (5.44–5.64)	7.19 (7.05–7.32)		
**LSI (*n* = 571)**					
Model 1	16.86 (15.57–18.24)	16.67 (15.43–18.01)	17.70 (16.41–19.10)	0.491	0.014 (−0.019; 0.047)
Model 2	17.10 (15.47–18.90)	16.91 (15.43–18.01)	17.70 (16.41–19.10)	0.486	0.011 (−0.023; 0.045)

VAT: Visceral adipose tissue; SAT: Subcutaneous adipose tissue; LSI: Liver signal intensity; BMI: Body mass index; IQR: Interquartile range; CI: Confidence Interval. Model 1: Adjusted for age and sex. Model 2 is model 1 but additionally adjusted for education, physical activity, smoking status, vitamin E supplementation, alcohol intake, and total energy intake. Model 3 is model 2 but additionally adjusted for BMI.

**Table 4:** Multivariable-adjusted means and 95% CI of VAT, SAT, and LSI according to tertiles of γ-tocopherol/cholesterol ratio, and scaled by IQR.

Outcome	Tertiles (T) γ-Tocopherol/Cholesterol Ratio			P_trend_	β Scaled by IQR and 95% CI
	T1	T2	T3		
N	196	199	196		
Median γ -tocopherol/cholesterol ratio (IQR), µmol/mmol	0.14 (0.13–0.14)	0.24 (0.23–0.25)	0.37 (0.36–0.39)		
**VAT, dm^3^ (*n* = 591)**					
Model 1	2.90 (2.68–3.15)	3.17 (2.92–3.44)	3.48 (3.21–3.78)	0.0002	0.073 (0.034; 0.111)
Model 2	2.92 (2.65–3.21)	3.21 (2.88–3.57)	3.45 (3.11–3.83)	0.0006	0.066 (0.027; 0.104)
Model 3	3.16 (2.97–3.37)	3.25 (3.02–3.49)	3.48 (3.24–3.73)	0.0034	0.037 (0.011; 0.063)
**SAT, dm^3^ (*n* = 591)**					
Model 1	5.80 (5.35–6.29)	6.40 (5.90–6.95)	6.65 (6.13–7.21)	0.006	0.059 (0.020; 0.099)
Model 2	5.81 (5.28–6.39)	6.51 (5.84–7.25)	6.58 (5.92–7.30)	0.011	0.048 (0.010; 0.087)
Model 3	6.36 (6.04–6.70)	6.61 (6.23–7.01)	6.64 (6.27–7.03)	0.103	0.015 (−0.006; 0.037)
N	190	191	190		
Median γ-tocopherol/cholesterol ratio (IQR), µmol/mmol	0.14 (0.14–0.15)	0.24 (0.23–0.25)	0.38 (0.36–0.39)		
**LSI (*n* = 571)**					
Model 1	16.32 (15.00–17.61)	17.41 (16.11–18.82)	17.60 (16.30–19.00)	0.193	0.017 (−0.020; 0.055)
Model 2	16.80 (15.35–18.40)	18.05 (16.30–20.00)	17.96 (16.28–19.82)	0.304	0.012 (−0.026; 0.051)

VAT: Visceral adipose tissue; SAT: Subcutaneous adipose tissue; LSI: Liver signal intensity; BMI: Body mass index; IQR: Interquartile range; CI: Confidence interval. Model 1: Adjusted for age and sex. Model 2 is model 1 but additionally adjusted for education, physical activity, smoking status, vitamin E supplementation, alcohol intake, and total energy intake. Model 3 is model 2 but additionally adjusted for BMI.

**Table 5:** Odds Ratio and 95% Confidence Interval for the association of α- and γ-tocopherol/cholesterol ratio with metabolic syndrome (MetS) and fatty liver disease (FLD).

**Outcome**	**Tertiles (T) of α-Tocopherol/Cholesterol Ratio**			**P_trend_**
	**T1**	**T2**	**T3**	
Median α-tocopherol/cholesterol ratio (IQR), µmol/mmol	4.63 (4.25–4.88)	5.53 (5.36–5.72)	6.74 (6.33–7.59)	
**MetS (yes/no) (258/383)**	(78/135)	(77/137)	(103/111)	
Model 1	Reference	1.01 (0.67–1.51)	1.72 (1.15–2.58)	0.006
Model 2	Reference	1.09 (0.72–1.65)	1.83 (1.21–2.76)	0.003
Median α-tocopherol/cholesterol ratio (IQR), µmol/mmol	4.61 (4.25–4.87)	5.52 (5.35–5.73)	6.75 (6.29–7.57)	
**FLD (yes/no) (224/347)**	(72/113)	(75/120)	(77/114)	
Model 1	Reference	1.03 (0.67–1.58)	1.11 (0.72–1.70)	0.631
Model 2	Reference	1.01 (0.65–1.55)	1.09 (0.70–1.68)	0.694
	**Tertiles (T) of γ-Tocopherol/Cholesterol Ratio**			**P_trend_**
	**T1**	**T2**	**T3**	
Median γ-tocopherol/cholesterol ratio (IQR), µmol/mmol	0.16 (0.13–0.18)	0.24 (0.22–0.26)	0.35 (0.31–0.41)	
**MetS (yes/no) (258/383)**	(70/143)	(89/125)	(99/115)	
Model 1	Reference	1.58 (1.05–2.39)	1.92 (1.28–2.89)	0.002
Model 2	Reference	1.50 (0.98–2.29)	1.87 (1.23–2.84)	0.004
Median γ-tocopherol/cholesterol ratio (IQR), µmol/mmol	0.16 (0.13–0.18)	0.24 (0.22–0.26)	0.34 (0.31–0.42)	
**FLD (yes/no) (224/347)**	(72/113)	(75/120)	(77/114)	
Model 1	Reference	0.99 (0.65–1.52)	1.38 (0.90–2.10)	0.124
Model 2	Reference	0.97 (0.62–1.51)	1.31 (0.85–2.02)	0.204

IQR: Interquartile range; MetS: Metabolic syndrome; FLD: Fatty liver disease model 1: adjusted for age and sex. Model 2 is model 1 but additionally adjusted for education, physical activity, smoking status, vitamin E supplementation, alcohol intake, and total energy intake.

## Supplementary Materials

The following are available online at www.mdpi.com/2072-6643/9/10/1143/s1, Table S1: Missing covariates information; Table S2: Odds ratio and 95% Confidence Interval for the association of α-tocopherol/cholesterol ratio with individual components of MetS; Table S3: Odds ratio and 95% Confidence Interval for the association of γ-tocopherol/cholesterol ratio with individual components of MetS; Table S4: Sensitivity analysis: multivariable-adjusted means and 95% CI of VAT, SAT, and LSI according to tertiles of α-tocopherol/cholesterol ratio, and scaled by IQR after excluding vitamin E supplement users; Table S5: Sensitivity analysis: multivariable-adjusted means and 95% CI of VAT, SAT, and LSI according to tertiles of γ-tocopherol/cholesterol ratio, and scaled by IQR after excluding vitamin E supplement users; Table S6: Sensitivity analysis: odds Ratio and 95% Confidence Interval for the association of α- and γ-tocopherol/cholesterol ratio with MetS and FLD after excluding vitamin E supplement users.
