# Supplementary material for: Association of Vitamin E Levels with Metabolic Syndrome, and MRI-Derived Body Fat Volumes and Liver Fat Content
Source: Nutrients. 2017 Oct 18;9(10):1143. doi: 10.3390/nu9101143 (PMC5691759; doi:10.3390/nu9101143)
Supplement: Supplementary file 1 [file nutrients-09-01143-s001.zip › nutrients-215788-supplementary.pdf]

Supplementary Materials

Table S1. Missing covariates information.

Covariate	Number of Missing Values
Cholesterol	1
Physical activity	1
Education (years)	1
Smoking status	8
Vitamin E supplementation	1

Table S2. Odds ratio and 95% Confidence Interval for the association of α-tocopherol/cholesterol ratio with individual components of MetS.

Outcome	Tertiles (T) of α-Tocopherol/Cholesterol Ratio			P_{trend}
	T1	T2	T3	
Median α-tocopherol/cholesterol ratio (IQR), μmol/mmol	4.63 (4.25–4.88)	5.53 (5.36–5.72)	6.74 (6.33–7.59)	
**Hypertension (yes/no) (135/506)**	(170/43)	(171/43)	165/49)	
Model 1	Reference	0.97 (0.59–1.60)	0.70 (0.42–1.16)	0.135
Model 2	Reference	0.99 (0.60–1.65)	0.71 (0.43–1.18)	0.145
**Low HDL-cholesterol (yes/no) (37/604)**	(7/206)	(9/205)	(21/193)	
Model 1	Reference	1.16 (0.41–3.25)	2.38 (0.95–5.99)	0.034
Model 2	Reference	1.25 (0.43–3.62)	2.52 (0.97–6.56)	0.033
**Hypertriglyceridemia (yes/no) (279/362)**	(29/184)	(29/185)	(96/118)	
Model 1	Reference	0.89 (0.50–1.58)	3.02 (1.81–5.04)	<0.0001
Model 2	Reference	0.91 (0.51–1.62)	3.02 (1.80–5.06)	<0.0001
**Hyperglycemia (yes/no) (129/512)**	(92/121)	(91/123)	(71/143)	
Model 1	Reference	0.99 (0.66–1.48)	0.97 (0.64–1.48)	0.888
Model 2	Reference	1.10 (0.72–1.66)	1.02 (0.67–1.57)	0.994
**Abdominal obesity (yes/no) (468/173)**	(148/65)	(161/53)	(159/55)	
Model 1	Reference	1.54 (0.96–2.42)	1.14 (0.72–1.82)	0.842
Model 2	Reference	1.42 (0.89–2.27)	1.13 (0.70–1.83)	0.834

IQR: Interquartile range; MetS: Metabolic syndrome. Model 1: Adjusted for age and sex. Model 2 is model 1 but additionally adjusted for education, physical activity, smoking status, vitamin E supplementation, alcohol intake, and total energy intake.

Table S3. Odds ratio and 95% Confidence Interval for the association of γ-tocopherol/cholesterol ratio with individual components of MetS.

Outcome	Tertiles (T) of γ-Tocopherol/Cholesterol Ratio			P_{trend}
	T1	T2	T3	
Median γ-tocopherol/cholesterol ratio (IQR), μmol/mmol	0.16 (0.13–0.18)	0.24 (0.22–0.26)	0.35 (0.31–0.41)	
**Hypertension (yes/no) (135/506)**	(166/47)	(167/47)	(173/421)	
Model 1	Reference	0.97 (0.59–1.57)	1.01 (0.61–1.68)	0.948
Model 2	Reference	0.92 (0.56–1.53)	0.97 (0.58–1.61)	0.907
**Low HDL-cholesterol (yes/no) (37/604)**	(4/209)	(14/200)	(19/195)	
Model 1	Reference	3.58 (1.13–11.33)	3.63 (1.18–11.15)	0.045
Model 2	Reference	3.95 (1.14–13.65)	4.67 (1.42–15.41)	0.018
**Hypertriglyceridemia (yes/no) (279/362)**	(33/180)	(37/177)	(59/155)	
Model 1	Reference	0.98 (0.58–1.68)	1.65 (0.99–2.73)	0.042
Model 2	Reference	1.06 (0.61–1.87)	1.81 (1.08–3.06)	0.014
**Hyperglycemia (yes/no) (129/512)**	(89/124)	(86/128)	(104/110)	
Model 1	Reference	0.92 (0.61–1.39)	1.25 (0.83–1.89)	0.249
Model 2	Reference	0.78 (0.51–1.21)	1.14 (0.74–1.75)	0.437
**Abdominal obesity (yes/no) (468/173)**	(141/72)	(159/55)	(168/46)	
Model 1	Reference	1.56 (1.00–2.45)	1.69 (1.07–2.69)	0.029
Model 2	Reference	1.57 (0.98–2.52)	1.59 (0.98–2.58)	0.065

IQR: Interquartile range; MetS: Metabolic syndrome. Model 1: Adjusted for age and sex. Model 2 is model 1 but additionally adjusted for education, physical activity, smoking status, vitamin E supplementation, alcohol intake, and total energy intake.

Table S4. Sensitivity analysis: Multivariable-adjusted means and 95% CI of VAT, SAT and LSI according to tertiles of α-tocopherol/cholesterol ratio, and scaled by IQR after excluding vitamin E supplement users.

Outcome	Tertiles (T) α-Tocopherol/Cholesterol Ratio			P_{trend}	β Scaled by IQR and 95% CI
	T1	T2	T3		
N	184	185	177		
Median α-tocopherol/cholesterol ratio (IQR), µmol/mmol	4.59 (4.24–4.83)	5.52 (5.35–5.71)	6.73 (6.35–7.57)		
**VAT, dm³ (*n* = 546)**					
Model 1	3.15 (2.95–3.38)	3.51 (2.28–3.76)	3.51 (3.27–3.76)	0.037	0.036 (−0.001; 0.074)
Model 2	3.06 (2.86–3.78)	3.42 (3.20–3.66)	3.42 (3.19–3.66)	0.026	0.037 (0.0004; 0.074)
Model 3	3.18 (3.03–3.33)	3.44 (3.28–3.60)	3.49 (3.33–3.65)	0.005	0.028 (0.003; 0.053)
**SAT, dm³ (*n* = 546)**					
Model 1	6.07 (5.67–6.50)	6.68 (6.24–7.16)	6.35 (5.92–6.80)	0.387	0.024 (−0.013; 0.062)
Model 2	5.95 (5.55–6.38)	6.49 (6.07–6.94)	6.20 (5.79–6.64)	0.423	0.024 (−0.012; 0.061)
Model 3	6.21 (5.97–6.46)	6.53 (6.29–6.78)	6.34 (6.10–6.59)	0.421	0.014 (−0.006; 0.035)
N	175	183	170		
Median α-tocopherol/cholesterol ratio (IQR), µmol/mmol	4.59 (4.24–4.86)	5.53 (5.35–5.73)	6.72 (6.29–7.48)		
**LSI (*n* = 528)**					
Model 1	18.64 (17.42–19.92)	18.79 (17.59–20.07)	18.83 (17.59–20.17)	0.825	0.003 (−0.023; 0.049)
Model 2	18.44 (17.24–19.84)	18.45 (17.25–19.73)	18.52 (17.26–19.87)	0.976	0.010 (−0.026; 0.046)

VAT: Visceral adipose tissue; SAT: Subcutaneous adipose tissue; LSI: Liver signal intensity; BMI: Body mass index; IQR: Interquartile range; CI: Confidence Interval. Model 1: Adjusted for age and sex. Model 2 is model 1 but additionally adjusted for education, physical activity, smoking status, alcohol intake, and total energy intake. Model 3 is model 2 but additionally adjusted for BMI.

Table S5. Sensitivity analysis: Multivariable-adjusted means and 95% CI of VAT, SAT and LSI according to tertiles of γ-tocopherol/cholesterol ratio, and scaled by IQR after excluding vitamin E supplement users.

Outcome	Tertiles (T) γ-Tocopherol/Cholesterol Ratio			P_{trend}	β Scaled by IQR and 95% CI
	T1	T2	T3		
N	167	193	186		
Median γ -tocopherol/cholesterol ratio (IQR), µmol/mmol	0.16 (0.13–0.18)	0.24 (0.22–0.26)	0.34 (0.31–0.41)		
**VAT, dm³ (*n* = 546)**					
Model 1	3.07 (2.87–3.29)	3.40 (3.18–3.64)	3.71 (3.47–3.98)	0.0001	0.079 (0.038; 0.120)
Model 2	3.03 (2.83–3.24)	3.32 (3.10–3.55)	3.59 (3.36–3.85)	0.0004	0.072 (0.032; 0.112)
Model 3	3.25 (3.11–3.41)	3.31 (3.16–3.47)	3.55 (3.39–3.72)	0.0072	0.0395 (0.012; 0.066)
**SAT, dm³ (*n* = 546)**					
Model 1	5.75 (5.37–6.15)	6.26 (5.85–6.70)	6.58 (6.15–7.04)	0.004	0.065 (0.024; 0.106)
Model 2	5.82 (5.44–6.22)	6.35 (5.93–6.80)	6.53 (6.10–7.00)	0.011	0.055 (0.015; 0.095)
Model 3	6.31 (6.08–6.56)	6.34 (6.10–6.58)	6.45 (6.20–7.00)	0.290	0.017 (−0.006; 0.040)
N	164	185	179		
Median γ-tocopherol/cholesterol ratio (IQR), µmol/mmol	0.16 (0.13–0.18)	0.24 (0.22–0.26)	0.34 (0.31–0.42)		
**LSI (*n* = 528)**					
Model 1	17.73 (16.55–19.00)	18.99 (17.79–20.26)	19.49 (18.24–20.83)	0.053	0.023 (−0.018; 0.064)
Model 2	17.72 (16.52–19.00)	18.86 (17.64–20.17)	19.20 (17.93–20.57)	0.096	0.018 (−0.022; 0.059)

VAT: Visceral adipose tissue; SAT: Subcutaneous adipose tissue; LSI: Liver signal intensity; BMI: Body mass index; IQR: Interquartile range; CI: Confidence Interval. Model 1: Adjusted for age and sex. Model 2 is model 1 but additionally adjusted for education, physical activity, smoking status, alcohol intake, and total energy intake. Model 3 is model 2 but additionally adjusted for BMI.

Table S6. Sensitivity analysis: Odds ratio and 95% Confidence Interval for the association of α- and γ-tocopherol/cholesterol ratio with MetS and FLD after excluding vitamin E supplement users.

Outcome	Tertiles (T) of α-Tocopherol/Cholesterol Ratio			P_{trend}
	T1	T2	T3	
Median α-tocopherol/cholesterol ratio (IQR), µmol/mmol	4.61 (4.24–4.87)	5.53 (5.36–5.72)	6.72 (6.34–7.55)	
**MetS (yes/no) (242/351)**	(74/127)	(74/126)	(94/98)	
Model 1	Reference	1.07 (0.70–1.63)	1.78 (1.17–2.71)	0.006
Model 2	Reference	1.15 (0.75–1.76)	1.84 (1.20–2.82)	0.004

Median α-tocopherol/cholesterol ratio (IQR), μmol/mmol	4.59 (4.24–4.86)	5.53 (5.35–5.73)	6.72 (6.29–7.48)	
**FLD (yes/no) (206/322)**	(67/108)	(71/112)	(68/102)	
Model 1	Reference	1.10 (0.71–1.71)	1.13 (0.72–1.77)	0.597
Model 2	Reference	1.09 (0.70–1.71)	1.12 (0.71–1.76)	0.628
	**Tertiles (T) of γ–tocopherol/cholesterol ratio**			
	**T1**	**T2**	**T3**	**P_{trend}**
Median γ-tocopherol/cholesterol ratio (IQR), μmol/mmol	0.16 (0.13–0.18)	0.24 (0.22–0.26)	0.35 (0.31–0.41)	
**MetS (yes/no) (242/351)**	(59/123)	(87/121)	(107/96)	
Model 1	Reference	1.60 (1.04–2.47)	1.99 (1.29–3.06)	0.002
Model 2	Reference	1.55 (1.00–2.41)	1.94 (1.23–3.00)	0.004
Median γ-tocopherol/cholesterol ratio (IQR), μmol/mmol	0.16 (0.13–0.18)	0.24 (0.22–0.26)	0.34 (0.31–0.42)	
**FLD (yes/no) (206/322)**	(61/103)	(67/118)	(78/101)	
Model 1	Reference	0.97 (0.62–1.51)	1.35 (0.87–2.11)	0.155
Model 2	Reference	0.93 (0.59–1.47)	1.32 (0.84–2.07)	0.196

IQR: Interquartile range; MetS: Metabolic syndrome; FLD: Fatty liver disease. Model 1: Adjusted for age and sex. Model 2 is model 1 but additionally adjusted for education, physical activity, smoking status, alcohol intake, and total energy intake.
